# Supplementary material for: Patterns of Intron Gain and Loss in Fungi
Source: PLoS Biol. 2004 Nov 30;2(12):e422. doi: 10.1371/journal.pbio.0020422 (PMC532390; doi:10.1371/journal.pbio.0020422)
Supplement: Table S1 — Also available at http://genes.mit.edu/NielsenEtAl/. (4.3 MB ZIP). [file pbio.0020422.st001.zip › NielsenEtAl/html/938.html]

AN9286.1.NCU07351.1.MG07646.1.FG03629.1


```
 CLUSTAL W (1.82) Multiple Sequence Alignments - Introns Inserted


Sequence 1: NCU07351.1	836 aa
Sequence 2: FG03629.1	843 aa
Sequence 3: AN9286.1	847 aa
Sequence 4: MG07646.1	863 aa
Alignment Length: 868 aa
Number Identitical Residues: 373 aa
Alignment Score (without introns) 19356


MG07646.1 	MMLYSLVLLGLAAIVRAEDGSAAWLRYAPLD--DAAAARFKAPDAIVALNSTQTSPVFTA
NCU07351.1	MRVSWGLLAFPAAASAIENGINGWLRYAPLPNNERPPNLR-LPGAIMALSTNKTSPVYTA
FG03629.1 	MRVSTLLLAFSTCVLAED-GLDGWLRYAPLPAHQVSSHAKSLPRNIRVIGGSKNSPLRSA
AN9286.1  	MRSFLLLTALLGVAAVAEDGLAAWLRYAPIP--HAKSYHKNLPSVIVPLNATAGRPIDTA
          	*     :          :.*  .******:   .  .   . *  *  :. .   *: :*

MG07646.1 	GQELQKGFNGMLGKNVNLVSSPEDLDPEDKDSRIIVVGTAAQFTDETTLGNLTFSPGDLE
NCU07351.1	GQELQAGIKGILNQTLPLYHEPDEW-----LGSTILVATTG----EFHGG---KPAATLA
FG03629.1 	ASELQKGFSGILNIDLKTADSKQTCD----FAKTIVVTTVDNLEKACHDK---QKHPKLE
AN9286.1  	AYELVDGIKGIFGKRVTLKNETRDDP----NLPAVTVGTVEAYAEAGGDV---SSVPELI
          	. **  *:.*::.  :    .             : * *.    .             * 

MG07646.1 	EDGFYMSTRGRVVQIVGQNERGALYGAFAYLARIAKHEFCGKDGIDNPHQPIRWVNQWDN
NCU07351.1	DDGYLLHVRPGNVVILGQTERGALYGAFDYLSRIAQGRFSDADLIANPDAPIRWVNQWDN
FG03629.1 	EDGYWLSTSDNSVKIIGQNERGALYGAFEYLSMLGQGNFKHVEKASNPSAPIRWANQWDN
AN9286.1  	DDGYYLSVAGPSVLILGQNERGALYGTFQYLERLAQGKVSDTSFASNPSAPIRWVNQWDN
          	:**: : .    * *:**.*******:* **  :.: ..   .   **  ****.*****

MG07646.1 	LDDTGTHGSVERGYAGPSIFFA-NGGVVADMTRAAEYARLLASIRINAVVVNNVNSNPQI
NCU07351.1	MDG-----SIERGYAGASIFFR-NNTIPKDLNRVSQYARLLASIGINGIVVNNVNANANL
FG03629.1 	LNASSTHGSIERGYGGASIFFDGIKNVRKDLSRVPLFGRLLASIGINAVVINNVNADVRL
AN9286.1  	LQDGGTHGSVERGYGGDSIFFW-DGRVRDDLTRASQYARLLASIGLNAVIVNNVNANETI
          	::  .: .*:****.* ****     :  *:.*.. :.****** :*.:::****::  :

MG07646.1 	LSPGNIDGLGRIADAFRPYGVQLGLSLNFASPQTYGGLDTFDPLDERVIKWWEDKTDELY
NCU07351.1	LTDANIDGLGRIADAMRPYGVQIGISLNFASPQTLGGLSTFDPLDRSVISWWTDKTNRLY
FG03629.1 	FNKENRDGLVEIADLLRPWGVQVGLSLNFASPSVTGDLDTFDPLDKKVIKWWHDLTDGLY
AN9286.1  	LTQENMDGVARIADAFRPYGIQLGLSLNFASPQSLGGLDTFDPFDERVISWWGEITDELY
          	:.  * **: .*** :**:*:*:*:*******.  *.*.****:*. **.** : *: **

MG07646.1 	ARIPDMAGYLIKANSEGQPGPHSYNR~TLAEGANLFAKATKHHGGLVMFRAFVYDH--LA
NCU07351.1	RRIPDMAGYLVKANSEGQAGPLTYNR~TLADGANLFAKAVQPHGGIVMFRAFVYDN--HI
FG03629.1 	ERIPDMAGYLVKANSEGQPGPLTYNR1TLADGANMFAKALEPHGGIVMFRAFVYD---KL
AN9286.1  	ERIPDMAGYLVKANSEGQPGPFTYNR~TLADGANLFARALQPHGGIVLFRAFVYDHENLN
          	 *********:*******.** :*** ***:***:**:* : ***:*:******* ..  

MG07646.1 	DWTNPKLDRAKAAVDYFEGLDEEWDDNVIVQIKYGPIDFQVREPPSPLFAHLPTTDTVIE
NCU07351.1	NPADLKADRAKHAVEFFKGLDGQFLDNVVVQIKYGPIDFQVREPASPLFANIPQTNTAIE
FG03629.1 	DWTDWKADRANAAVEFFSELDGKFDDNVVVQIKYGPIDFQVREPVSPLFSHLRNTNSAIE
AN9286.1  	ETLDWKADRANAAVEFFDGLDPQFEDNVVIQIKNGPIDFQVREPVSPLFAHLSQTASAVE
          	:  : * ***: **::*. ** :: ***::*** ********** ****:::  * :.:*

MG07646.1 	LQVTQEYLGQQNHLVYMAPLWKEILGFDLHIEGQEKSLVRDVVAGERPGGKKLRLSGSAA
NCU07351.1	LQITQEYLGQQSHLVYLAPLWETILNFDLRADDQ-PSLVRDVVAGHRFSNK---RGGYAG
FG03629.1 	LQISQEYLGQQCHLVYLPPLWRTILDFDMRIDGK-KSNVRDIVSGKVFKRK---LGGYAG
AN9286.1  	LQVTQEYLGQQCHLVYLAPMWKEVLDFDLRVDGK-DSVVSDIVSGRRFNNT---LGGYAG
          	**::******* ****:.*:*. :*.**:: :.:  * * *:*:*.    .    .* *.

MG07646.1 	VVNVGTDANWLGSHLAMSNLYAYGVLAWDPMADSEAMLRDWIKLTFGHDQLVADTIVKMS
NCU07351.1	VVNVGTNTTWLGSHLAMSNLYAYGRLAWNPNQDPQALLEDWTRLTFGSDPLVLGVVTEIS
FG03629.1 	VTNVGLDKTWLGSHLSMSNLYAFGRLAWNPQADPQAVLEDWTRLTFGLDNKVRNAITEMS
AN9286.1  	VVNVGLNTTWLGSHLAMSNLYAYGRLAWDPSADSVELLQEWIKMTFSHDQEVVDVITKMS
          	*.*** : .******:******:* ***:*  *.  :*.:* ::**. *  * ..:.::*

MG07646.1 	MESWPAYEDYSGNLGIQTLADILHTHFGPGPQSQDGNGWGQWTRANATSVGMDRTVATGT
NCU07351.1	MASWPAYENYTGNFGIQTLTDILYTHYGPNPASQDNNGWGQWTRADHNGIGMDRTVSTGT
FG03629.1 	MESWPAYENYSGNLGIQTLTDILYAHYGPNPASQDNNGWGQWTRATRDHIGMDRTVKNGT
AN9286.1  	MESWPAYENYSGNLGIQTLTDILLGHYGPNPASQDGNPWGQWTRADADSIGMDRTVWNGT
          	* ******:*:**:*****:***  *:**.* ***.* *******    :****** .**

MG07646.1 	GFSGQYPPEVAQRFEKIETTPDNLILWFHHVPWTHVLKSGKTVIQHFYDAHYDGSALVQE
NCU07351.1	GFSGQYPAEVAEKFESIETTPDDLLLWFHHVNYTHVLKSGKSVIQHFYDAHYAGAETAQT
FG03629.1 	GNAGQYPAEVAERFENPDTTPDDLMLWFHHVPYDFKLHSGKTVIQHFYDAHYKGANTAQT
AN9286.1  	GNAGQYPEEVYQMYENIDTTPDNLLLWFHHVPYTQRLKSGKTVIQHFYDAHYRGSATAQT
          	* :**** ** : :*. :****:*:****** :   *:***:********** *:  .* 

MG07646.1 	FPLQWRALQGHMDDERFEHMLFRLNFQAGHALVWRDSICNFYNNMTSIPDEKGRVGNHPY
NCU07351.1	FPIAWESLRNKIDTERFNDVLFRLRYQAGHSIVWRDSICQFYYNLSGIPDSTGRVGKHEY
FG03629.1 	FPKLWKSLQGKVDEDRFKHELFRLNYQAGHSIVWRDAISEFYRNLSGIADTQDRVRNHPY
AN9286.1  	FVSLWKTIKGKIDKERYEHVLFRLVYQAGHALVWRDSITNFYYNKSGIPDEAGRVGNHPY
          	*   *.:::.::* :*::. **** :****::****:* :** * :.*.*  .** :* *

MG07646.1 	RIEAENMEMEGYRVYNVDPFHMASARRAVITTSNTTTGANSTDPGTVWATLDFEDGIYDV
NCU07351.1	RIEAEAMALKNYKLTGVNPVETASNLTAVVLVSNHTSGEATTK-------LNFDSGKYDV
FG03629.1 	RIEAEKMDLDNYHPVNVTPTETASKYVAVYTNG---TGTATTK-------LKFKKGTYNL
AN9286.1  	RIEAEDMELDGYEPYLVSPFEAASGSHCIVTSNNSTEGRASTP-------LKVKNGKYDI
          	***** * :..*.   * * . **   .:   .. : *  :*        *....* *::

MG07646.1 	AVNYYDVMRGQADYVLTIDNKVIGRWRGDNEDRLGNTGTLNLDGHSATRIRFENIDIKKG
NCU07351.1	TINYFDLIGGKAHYKAFLNGKLLLEWAGDLEDKLGHTFSTKLDGHSATRVTIRDVQIQKG
FG03629.1 	AVNYYDVARGKATWAVYLNKRLVGKWIGDNEEKLGHWPSDFLDGHSATRMTFEGVKIKPG
AN9286.1  	AVNYFDQAIGNSTWRLFLDDDLVGEWKGDLEYILGRAPSPYIDGQTAARITFKHVHIKSR
          	::**:*   *:: :   ::  :: .* ** *  **.  :  :**::*:*: :. :.*:  

MG07646.1 	DVLRIVGTPDGIEGAPLDYVAILPDGIVD
NCU07351.1	DVLKITGVADGNELAPLDYIAVLPQGVVD
FG03629.1 	DELTIIGKADGAEKAALDYISLLPEGVVD
AN9286.1  	STLSIVGIPDGMEPAPIDYVSILPEGVID
          	. * * * .** * *.:**:::**:*::*
```
